# Making the world from topological order

**DOI:** 10.1093/nsr/nwy116

**Published:** 2018-11-06

**Authors:** Philip Ball

**Affiliations:** writes for NSR

## Abstract

Topology used to be a term confined to a branch of pure mathematics, where it referred to an invariant property of shape. The classic example was the way objects containing a single hole, like a torus and a coffee cup with handle, can be smoothly moulded into one another without tearing. But topological considerations have long played a role in the physics of matter, where for example they might dictate particular arrangements of component parts that can’t be erased from the system. The classic example here is the fact that a ‘hairy ball’ can’t be combed flat without having at least two pointy tufts. Such ‘defects’ in organization can be considered ‘topologically protected’, since they are robust against any recombing of the hair. They are universal features that don’t depend on the material specifics of the system: topological defects in liquid crystals are analogous to defects in spacetime called cosmic strings.

In the past several decades in particular, properties of matter arising from topological considerations have become a major theme, reflected for example in the award of the 1985 and 1998 Nobel Prizes in Physics for discoveries involving the quantum Hall effect. Here the ‘Hall conductance’, quantifying the passage of electrical current in a 2D conductor in the presence of a transverse magnetic field, takes precise integral or fractional multiples of a particular quantized value related to the electron charge. This behaviour persists regardless of how we modify the material, for example by adding impurities. Topological phases and transitions were also a feature of the work that won the 2016 Nobel Prize.

It has become recognized that the topological properties of the quantum-mechanical electronic structures of certain materials can give them unusual and perhaps useful properties. Some researchers think, for example, that ‘topological matter’ might supply quantum bits for quantum computation that resist the randomizing effects of noise.

Xiao-Gang Wen of the Massachusetts Institute of Technology has been developing ideas about ‘topological order’ in fundamental physics for several decades. His notions of how topology in the underlying structure of spacetime might give rise to fundamental particles and forces make a connection to the topological phases recognized in condensed matter, revealing a new unifying principle in physics. National Science Review spoke to him about his work.

**NSR:** Could you explain what ‘topological order’ means in states of matter? It seems to relate to very deep concepts in physics, such as symmetry and its breaking, that apply as much to fundamental particle physics as they do to the behaviour of tangible and useful materials.

**Wen:** Many different phases of matter exist in nature. According to the theory of phase transitions by Russian physicist Lev Landau, the reason phases are different is because they have different symmetries in the organization of their constituent particles. For a long time, people believed that Landau's theory described all phases of matter, creating a feeling that the theory of condensed matter was complete.

However, the study of phases called chiral spin liquids and quantum Hall liquids, in which quantum-mechanical effects dominate, in the 1980s revealed that these states all have exactly the same symmetry. Their difference cannot be understood in terms of symmetry. Rather, there is a new organizational principle that sets them apart: topological order. Thus topological order describes phenomena that cannot be characterized by symmetry and its breaking.

Although the theory of topological order was invented to understand new phases of quantum matter, it may also describe the internal structure of empty space. This is because space may be viewed as a system formed from many quantum bits (qubits). A qubit is the basic unit of quantum information, and can have two values: say, 0 and 1. So we may view space as a qubit ocean. When such a qubit ocean has a proper topological order—that is, when the qubits organize in a certain way—their fluctuations, and defects in their topological order, can produce photons and electrons respectively, as well as all other elementary particles. In this way we obtain a unification of forces (which physicists call gauge interactions) and matter. This unification is even more profound than the so-called grand unification, which just unifies different gauge interactions (forces). It brings together interaction, matter and information, while suggesting that the essence of our universe is quantum information. Matter and interactions all come from quantum information.

Matter and interactions all come from quantum information.

—Xiao-Gang Wen

**Figure 1:**
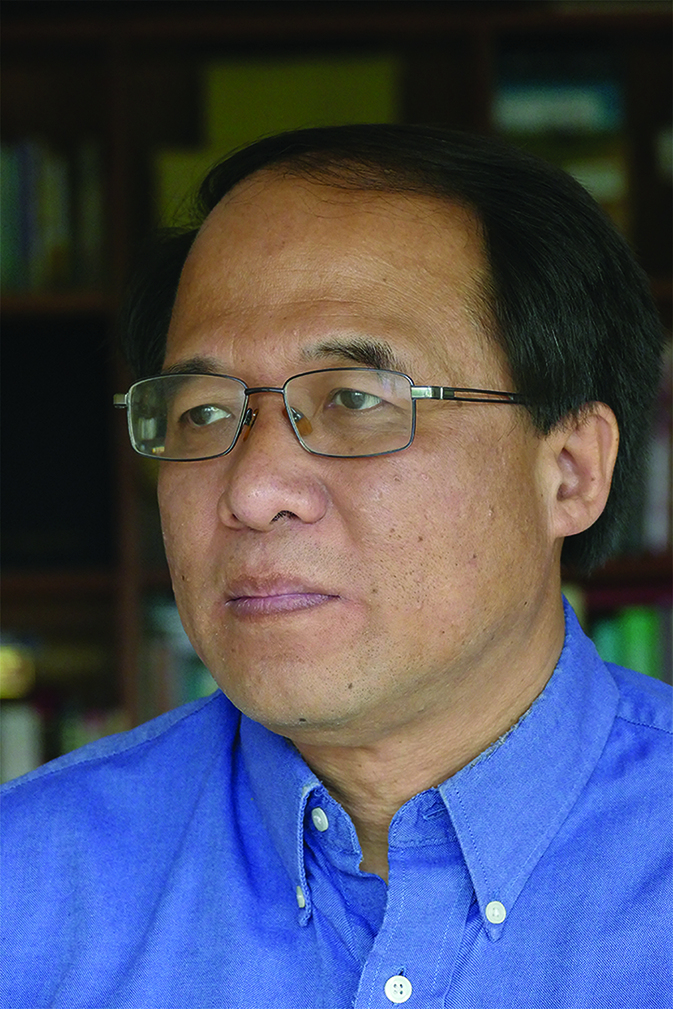
Professor Xiao-Gang Wen of the Massachusetts Institute of Technologyhas been developing ideas about ‘topological order’ for several decades *(Courtesy of Xiao-Gang Wen)*.

**NSR:** The idea that topological order plays an important role in some phases of condensed matter has become a huge field of research over the past decade or two. How and when did this start to emerge within physics?

**Wen:** After the experimental discovery of the fractional quantum Hall effect in the early 1980s, people realized that the exotic ‘liquids’ that show this effect have many unusual properties, such as fractional charges and fractional statistics. This indicates that fractional quantum Hall liquids are new states of matter. But what is their essence? In 1989, while studying related chiral spin liquids, I realized that the essence of the new states of matter has nothing to do with symmetry. One needs to use a totally different point of view, and the new angle I tried was topology, motivated by Ed Witten's work on topological quantum field theory.

This approach works perfectly. I found that the new kinds of organization could be characterized by the different ground-state degeneracies [the number of lowest-energy states] as we put the new state of matter into spaces with different topologies. I conjectured that the so-called non-Abelian geometric phases from these degenerate ground states can fully characterize and define the new order, which I named topological order. A year later, I showed that the boundaries of fractional quantum Hall liquids have robust properties, reflecting the different topological orders carried by these systems in the bulk region. This allows us to measure topological order in experiments.

The theory of topological order is very rich and deep. Starting in 1989, I have been working on topological order for almost 30 years, and am still making new discoveries. Around 2000, a deep connection was discovered between topological order and quantum entanglement, and an application of topological order to quantum computation was proposed by Alexei Kitaev. So in the last 20 years, the field of topological order has enjoyed steady growth, and has now become one of the major fields in condensed-matter physics and quantum computation.

**NSR:** You see topological order as something of a unifying principle to understand the states of matter. How does that work?

**Wen:** To understand states of matter at low temperatures, we need both Landau's notion of symmetry breaking and the notion of topological order. Symmetry breaking describes the static pattern in the organization of the constituent particles. However, the particles can also have so-called quantum fluctuations, which also have patterns. Topological order describes these ‘dancing’ patterns, which are patterns of quantum entanglement in a quantum material.

**NSR:** You have proposed something called ‘string-net condensation’ as a deep principle in quantum field theory. Can you explain what this is?

**Wen:** There is a principle of emergence in condensed-matter physics: the properties of matter come from the internal organization of its parts. It's the same for space, viewed, as I said above, as a special piece of matter formed by qubits. In this view, deformations in the patterns of qubits are light waves that cause the electromagnetic interaction, and gluons that cause the strong interaction. The internal order can also have defects corresponding to electrons and other matter particles, such as quarks.

Light waves, gluons, electrons and quarks can thus be regarded as properties of space, viewed as a qubit ocean. We know that for our space, light waves satisfy the Maxwell equations, gluons satisfy the Yang–Mills equation, and electrons and quarks satisfy the Dirac equation. But only when the space has a particular topological order—when the qubits that form the space are organized in a certain way—can this be so. String-net condensation is a term to describe this kind of organization. In this case, the value-1 qubits form a network of strings joined in a certain way. Otherwise, the strings can move around and reconnect freely via the flipping of qubits—that is, their value can change between 0 and 1. In such a string-net liquid, the density waves of the strings correspond to light and gluon waves, and the ends of the strings correspond to electrons and quarks. So my claim is that qubits with this string-net organization unify all elementary particles and interactions, and can provide an origin of the Standard Model of particle physics.

I have been working on topological order for almost 30 years, and am still making new discoveries.

—Xiao-Gang Wen

**NSR:** There are now many examples of materials that exhibit some kind of topological features in their electronic structure, from quantum spin liquids to Weyl fermions, graphene and topological insulators. These are exotic materials that are not easy to describe, but can you say what it is about them that pertains to topology, and how this manifests in the behaviours we can actually observe and measure?

**Wen:** In recent years, many very different phenomena have been called ‘topological’, which causes some confusion. In an article called ‘Zoo of quantum-topological phases of matter’, I tried to clarify some of this terminology [http://www.arxiv.org/abs/1610.03911].

Quantum spin liquids and quantum Hall liquids have topological order since they have special properties that are robust against any local perturbations, including perturbations that break all their symmetry. For example, in a certain string-net liquid, the emergent photons can carry arbitrarily small energies and cause the long-range Coulomb [electrostatic] interaction. We cannot destroy this property regardless of how we change the interactions between the underlying qubits. Thus the emergent long-range Coulomb interaction is a topological property. Robustness against any local perturbations is what ‘topological’ implies here.

Topological insulators are quite different. Their properties are not robust against some local perturbations, such as perturbations that break some symmetries, and thus are not ‘topological’ in that same sense. The fundamental difference is that topological insulators have only short-range quantum entanglement, while topological order has long-range entanglement, which is the source of topological robustness.

As for so-called topological superconductors, some of them have topological order and others do not. p-wave superconductors in one dimension and chiral p-wave superconductors in two dimensions do, but topological superconductors with time-reversal symmetry don’t. Meanwhile, the materials called Weyl semimetals, and also graphene, have emergent gapless fermions—they have electronic states for which the gap between the conduction and valence bands can fall to zero at certain points in momentum space. This property is protected by a combination of symmetry and topology.

Topological insulators have unusual boundary properties. Many articles say that topological insulators are characterized by being conducting at their edges. This description is not very accurate, since even non-topological insulators can have conducting edges. What is special about topological insulators is that their conducting boundary is not affected by impurities that preserve the time-reversal symmetry.

So we see that those materials are very rich and have a variety of properties. Some have topological order, and some have other types of order. Just using the term ‘topological’ is inadequate to capture their essence.

**NSR:** Do these materials have any potential uses that follow from the topological features of their electronic states?

**Wen:** Long-range-entangled topologically ordered materials, such as fractional quantum Hall liquids and p-wave topological superconductors, can be used to make topological quantum computers. The perfectly conducting boundary of topologically ordered materials may also find some applications in devices. However, currently topologically ordered materials exist only at very low temperatures or in very strong magnetic fields. For useful applications, we need to find new topologically ordered materials that exist at high temperatures and in weaker magnetic fields.

Short-range-entangled topological insulators have been realized at room temperature and zero magnetic field, and their symmetry-protected conducting boundary may find some device applications.

**NSR:** One of the great attractions of this field is that it seems to offer a model of how physics really works, as opposed to how many people outside the field think it works. That is to say, it often deals not in terms of specific kinds of systems or materials, but with concepts such as symmetry breaking, criticality, disorder and now topological order that apply to many different systems and scales. Is this how you see physics?

**Wen:** The development of topological order is quite different from some other developments in physics. Many of those developments are about the discovery of a particular new material or phenomenon. Those kinds of discoveries are certain hugely important. But the development of topological order is more about the discovery of a new way of thinking and a new way to view our world.

In a sense, Landau symmetry-breaking theory gives us the ability to hear and enjoy the wonderful ‘music’ that nature offers us. Likewise, topological order gives us the ability to see the wonderful ‘sights’ that nature offers us. Once our eyes are opened, we can discover many new beautiful pictures.

**NSR:** Do you see a strong interest in these questions of topological matter within China? If so, where/who are the prime movers?

**Wen:** Physicists and students in China have a very strong interest in topological matter, probably even more than other countries. On the theory side, Tao Xiang's group (Institute of Theoretical Physics, Chinese Academy of Sciences) has developed a sophisticated tensor-network approach, which is well suited to the numerical study of topological order. Zheng-Yu Weng (Tsinghua University) has developed a gauge-theory approach to spin liquids and has trained a team of world-class PhD students who become a driving force for studying topological states of matter. Chinese experimentalists have also made some great discoveries. Qi-Kun Xue's group (Tsinghua University) has discovered anomalous quantum Hall effects, and Jing-Feng Jia's group (Shanghai Jiao Tong University), as well as Hong-Jun Gao and Hong Ding (Institute of Physics, Chinese Academy of Sciences), have found evidence of Majorana zero modes [a fractional degree of freedom carried by a kind of topological quasi-particle]. In the areas of topological matter and iron-based superconductors, Chinese physicists are working at the frontier and are part of the global driving force.

**NSR:** How did you become interested in these matters? What or who inspired you to study them? And how would you attract young researchers to the field?

**Wen:** My interest in topological order is driven by my curiosity, not by its potential application—it has no predetermined goal and purpose. In basic scientific research, we try to expand the territory of our knowledge and discover the unknown. The unknown does not even have a name, and so cannot be the goal or the purpose of my research. So my research is mainly guided by what I think is beautiful. If I like it, I just do it, regardless of what other people think. The important thing is that it is the frontline researcher, not the government, who decides what is beautiful and leads the development of the field. This, at least, is how basic scientific research should be conducted. For applied research, it is industry that determines what is useful and directs the development.

My research is mainly guided by what I think is beautiful.

—Xiao-Gang Wen

I try to attract young researchers to my field by showing them the beauty that I see, and by enjoying those beautiful ‘pictures’ together with them.

## Notes

Philip Ball writes for NSR from London.

